# Mindfulness-based Cognitive Therapy (MBCT) in bipolar disorder: Preliminary evaluation of immediate effects on between-episode functioning

**DOI:** 10.1016/j.jad.2007.08.022

**Published:** 2008-04

**Authors:** J.M.G. Williams, Y. Alatiq, C. Crane, T. Barnhofer, M.J.V. Fennell, D.S. Duggan, S. Hepburn, G.M. Goodwin

**Affiliations:** University of Oxford, Department of Psychiatry, Warneford Hospital, Warneford Lane, Headington, Oxford, OX3 7JX, UK

**Keywords:** Bipolar disorder, Unipolar depression, Mindfulness-based Cognitive Therapy, Anxiety, Remission

## Abstract

**Background:**

Bipolar disorder is highly recurrent and rates of comorbidity are high. Studies have pointed to anxiety comorbidity as one factor associated with risk of suicide attempts and poor overall outcome. This study aimed to explore the feasibility and potential benefits of a new psychological treatment (Mindfulness-based Cognitive Therapy: MBCT) for people with bipolar disorder focusing on between-episode anxiety and depressive symptoms.

**Methods:**

The study used data from a pilot randomized trial of MBCT for people with bipolar disorder in *remission,* focusing on between-episode anxiety and depressive symptoms. Immediate effects of MBCT versus waitlist on levels of anxiety and depression were compared between unipolar and bipolar participants.

**Results:**

The results suggest that MBCT led to improved immediate outcomes in terms of anxiety which were specific to the bipolar group. Both bipolar and unipolar participants allocated to MBCT showed reductions in residual depressive symptoms relative to those allocated to the waitlist condition.

**Limitations:**

Analyses were based on a small sample, limiting power. Additionally the study recruited participants with suicidal ideation or behaviour so the findings cannot immediately be generalized to individuals without these symptoms.

**Conclusions:**

The study, although preliminary, suggests an immediate effect of MBCT on anxiety and depressive symptoms among bipolar participants with suicidal ideation or behaviour, and indicates that further research into the use of MBCT with bipolar patients may be warranted.

## 1 Introduction

Pharmacological treatment has been the treatment of choice for patients with bipolar disorder for many years. However, even with good maintenance medication, 73% of patients relapse within 5 years (Gitlin et al., 1995). Poorer overall outcome is associated with a high rate of comorbidity (Sasson et al., 2003), particularly with anxiety disorders, which are present in approximately 64% of bipolar patients (Simon et al., 2005). Therefore, psychological interventions have assumed increasing interest as a way of improving mood stability. Cognitive Behaviour Therapy (CBT) has had the clearest claims to be a specific intervention based broadly on Beck's ideas about how cognitions shape mood (Scott and Colom, 2005). However, initially promising findings (Lam et al., 2003) have not been confirmed in what was intended to be a definitive trial (Scott et al., 2006).

Recently, our group has been investigating the usefulness of a new psychological treatment for patients with a history of suicidality. Mindfulness-based Cognitive Therapy (MBCT) combines aspects of cognitive therapy with training in meditation. MBCT teaches people skills that enable them to become more aware of their thoughts without judgment, viewing negative (positive and neutral) thoughts as passing mental events rather than as facts. MBCT has proven effective in preventing relapse in recurrent depression (Ma and Teasdale, 2004; Teasdale et al., 2000). The treatment is closely based on an approach that is known to be helpful in the treatment of anxiety disorders (Mindfulness-based Stress Reduction: Miller et al., 1995), so this study provides an opportunity to evaluate its impact on between-episode anxiety in bipolar patients.

Our research has been concerned with the application of MBCT to those patients who become suicidal when depressed. Because bipolar disorder carries a high risk of suicide, we included patients with this diagnosis in a randomized controlled trial we were conducting. It has been estimated that between 10% and 15% of hospitalized bipolar patients die from suicide (Hawton et al., 2005) and the risk factors include depression and anxiety comorbidity. This suggests that therapeutic efforts to target any of these correlates might have an impact on the illness morbidity (Leverich et al., 2003) and perhaps mortality.

In summary, this study aimed to explore the feasibility and potential benefits of MBCT for people with bipolar disorder, using data collected from a pilot randomized controlled trial of MBCT for people with a history of suicidal ideation or behaviour. The study hypothesis was that MBCT would improve between-episode anxiety and depressive symptoms.

## 2 Method

### 2.1 Sample

The sample was recruited as part of preliminary randomized controlled trial that aimed to examine the acceptability and mechanisms of action of Mindfulness-based Cognitive Therapy (MBCT) delivered to patients in remission, but with a history of serious suicidal ideation or behaviour. Participants aged between 18 and 65 who met inclusion criteria (at least one prior episode of major depression accompanied by serious suicidal ideation) were recruited from general practitioners and local psychologists/psychiatrists and from the community. All participants were required to meet NIMH criteria for recovery at the time of participation (no more than one week of minimal depressive symptoms in the past 8 weeks), and to have experienced no manic episodes for at least 6 months.

One hundred and twenty eight people who contacted the group on time and were interested in the study were screened; only 83 were eligible. A further 15 participants did not attend their first assessment or withdrew after the first assessment. The final sample included 68 participants who were randomly allocated (concealed from trial team) to immediate treatment with MBCT (24 unipolar, 9 bipolar) or to waiting list control condition (27 unipolar, 8 bipolar). Follow-up data were available from 21 unipolar and 7 bipolar participants in the MBCT group and from 20 unipolar and 7 bipolar participants in the waitlist group.

### 2.2 Procedure

Participants were interviewed using the Mini International Neuropsychiatric Interview MINI (Sheehan et al., 1998) to establish psychiatric history. Following this, participants completed a number of other assessment measures. These measures were completed again at the end of treatment or waitlist; those of relevance to the current study are described below. Randomization was concealed by use of sealed envelopes prepared by an individual outside the research team.

### 2.3 Questionnaires

#### 2.3.1 Beck Depression Inventory (BDI-II) (Beck et al., 1996)

The BDI-II is a well established 21-item self report questionnaire used to measure the severity of depressive symptoms.

#### 2.3.2 Beck Anxiety Inventory (BAI) (Beck and Steer, 1990)

The BAI is a 21-item self report questionnaire that measures the common symptoms of anxiety. The scale is reliable and valid and is able to differentiate anxiety from depression.

### 2.4 Intervention

During the trial, both groups of patients were encouraged to continue to attend outpatient appointments, receive medication and benefit from any other services they would otherwise have received. At post-assessment participants were interviewed about treatment received during their period of involvement with study. In the control group 4 participants had received other psychotherapy by visiting their psychiatrists (3 unipolar, 1 bipolar), whilst in the MBCT group 4 participants had done so (2 unipolar, 2 bipolar). None of the participants reported using non-MBCT meditation or yoga for therapeutic purpose during their trial period.

The MBCT classes were led by two experienced therapists with recognized expertise in the delivery of cognitive therapy and MBCT. The classes included 12–15 participants and met weekly for 2 h over a period of 8 weeks, including a full-day of meditation practice following week 6. Participants were also asked to complete homework (including daily meditation practice, observations of thoughts, feelings and bodily reactions) which was expected to take at least 45 min per day, 6 days per week. The MBCT approach has been described in detail elsewhere (Segal et al., 2002).

## 3 Results

### 3.1 Sample characteristics

In these analyses we aimed to explore whether bipolar patients differed from unipolar patients in their baseline characteristics. We aimed also to explore whether bipolar in the MBCT differs from bipolar in waitlist group. Statistical analysis revealed no significant differences in the sample characteristics on most aspects, including gender, age and current occupation status.

### 3.2 Depression (BDI)

There were no main effects of *group* (*Unipolar*, *Bipolar*) or *condition* (*MBCT*, *waitlist*)*,* but there was a main effect of *time*, *F* (1, 41) = 6.07, *p *= .018, and a significant two-way *time* × *condition* interaction, *F* (1, 41) = 8.05, *p *= .007. Follow-up Bonferroni-corrected post-hoc comparisons indicated that patients receiving MBCT showed reduced depression at time 2 (*Mi-j* = − 1.29, SE = .34, *p* < .001) compared to time 1, while no time difference was found among patients in the waitlist condition. This resulted in a significant difference in level of depression between participants allocated to MBCT and those allocated to waitlist at time 2 (*Mi-j* = − 1.12, SE = .55, *p* < .05), whilst there was no significant difference at time 1. The three-way *time* × *group* × *condition* interaction was not significant.

### 3.3 Anxiety (BAI)

There were no main effects of *time*, *group* or *condition* on BAI scores. There were also no *group* × *time* or *group* × *condition* interactions. However there was a two-way *time* × *condition* interaction, *F* (1, 41) = 5.63, *p *= .022; and a three-way *time* × *group* × *condition* interaction, *F* (1, 41) = 7.55, *p *= .009.

Follow-up Bonferroni-corrected post-hoc comparisons found no difference in BAI level between bipolar patients in the waitlist and in the MBCT conditions at time 1. However, those who had received MBCT had significantly lower anxiety at time 2 than those in the waitlist condition (*Mi-j* = − 1.951, SE = .758, *p* = .014). Unipolar groups (MBCT and waitlist conditions) did not differ in BAI score at either time point. The comparisons also indicated that whilst bipolar patients receiving MBCT showed no significant change in BAI level from time 1 to time 2, bipolar patients in the waitlist condition showed a significant *increase* in their scores over time (*Mi-j* = 1.28, SE = .417, *p* = .004). No significant difference was found between time 1 and time 2 among unipolar patients. Means and standard deviations are shown in Table 1.

## 4 Discussion

This study reports data from a pilot randomized controlled trial of MBCT for patients with a history of suicidal ideation or behaviour (including unipolar and bipolar disorder). As far as we know, it is the first to assess the immediate benefits of MBCT for people with bipolar disorder. The results suggest significant effects of MBCT in limiting increased anxiety over time which were specific to the bipolar group. The effect of MBCT in reducing depression was observed among all participants attending MBCT (bipolar and unipolar) and was not specific to either group.

### 4.1 BAI measure

Anxiety disorders are highly comorbid with bipolar disorder and this comorbidity is associated with poor outcome and suicide risk. Hence anxiety symptoms are an important, if often neglected, indicator of treatment outcome (Goodwin and Sachs, 2004). It is thus of great potential interest in this study that the bipolar patients who received MBCT had significantly lower anxiety scores at time 2 compared with the bipolar waitlist controls. The unipolar group showed no such differential change in BAI score. This suggests that the protective effect of MBCT on levels of anxiety was specific to bipolar patients.

### 4.2 BDI measure

Depressive symptoms were significantly improved by MBCT for both unipolar and bipolar patients at time 2, and no differential effect was found for bipolar versus unipolar patients. The finding that MBCT reduces residual symptoms of depression is comparable with the pilot study of Scott et al. (2001) on cognitive therapy, which found that the bipolar patients who received cognitive therapy achieved significant reductions in level of depression as measured by the Internal State Scale.

Studies from different patient groups have shown that meditation-based therapy results in immediate post-treatment improvements in anxiety and depressive symptoms (Kabat-Zinn et al., 1992) and that this improvement is maintained over 3 years follow-up (Miller et al., 1995). Studies also show that patients with three or more past episodes of unipolar depression who receive MBCT have lower relapse rates over a 12-month follow-up than waitlist controls (Ma and Teasdale, 2004; Teasdale et al., 2000). The current study suggests that MBCT improves anxiety and depressive symptoms in bipolar patients. It clearly suggests that MBCT may be a trans-diagnostic therapeutic tool, suitable for bipolar disorder as well as other psychiatric conditions.

## 5 Limitations

The study suffers from a number of limitations that restrict the confidence with which conclusions can be drawn. First, it is preliminary and was designed to establish feasibility and acceptability. The sample sizes are small. Moreover, the study was not designed specifically to demonstrate a difference between bipolar and unipolar patients and so did not include measures of manic experience or symptoms. It is possible that some individuals had experienced subsyndromal manic/hypomanic symptoms during the study period. Further trials on MBCT in bipolar disorder should use measures of manic symptoms, or mania-related beliefs (e.g. the HAPPI: Mansell, 2006).

Secondly, some psychological treatments for bipolar disorder may work partly through increasing medication adherence or producing changes in life style that encourage greater regularity (Bauer, 2002). We cannot rule out such treatment effects due to non-specific group factors. However, there is a reason to expect specific benefits from MBCT in bipolar disorder because it provides a mood regulation strategy in its own right. It teaches people to notice mood shifts early and to accept them rather than strive to avoid them, and thus may help to prevent the escalation of mood-activated patterns of unhelpful thinking.

Finally, the study preferentially recruited patients with a history of suicidal ideation or behaviour as part of their symptom profile, so we cannot immediately generalize the findings of this study to those patients who do not show such symptoms. However, patients who habitually become suicidal when depressed are often the most difficult to treat, and create the greatest concern amongst clinicians. Finding significant changes in important areas of psychopathology in this sub-group is very promising. However, future research will need to include a follow-up to confirm whether these short-term effects will persist, and be related to a less severe illness course.

In summary, suicidal bipolar and unipolar patients show reductions in depressive symptoms following treatment with MBCT. Intriguingly, the largest effects – on anxiety – were confined to the bipolar group. These initial results are only preliminary but are sufficient to suggest that further investigation of MBCT for bipolar disorder is warranted.

## Role of funding source

This study was supported by the Wellcome Trust (GR067797). The Wellcome Trust had no further role in the study design; in the collection, analysis and interpretation of data; in the writing of the report; or in the decision to submit the paper for publication.

## Conflict of interest

None of the authors has any conflicts of interest with respect to this paper.

## Figures and Tables

**Table 1:** Measures of mood

Measures of mood	Unipolar				Bipolar			
	Control (20)		MBCT (14)		Control (7)		MBCT (7)	
	Pre	Post	Pre	Post	Pre	Post	Pre	Post
BAI	9.3 (6.5)	8.0 (5.8)	10.7 (8.6)	10.7 (10.5)	11.4 (8.5)	20.6 (11.3)	12.7 (12.1)	6.8 (5.7)
BDI	12.8 (10.2)	11.2 (10.8)	15.5 (14.1)	9.5 (13.8)	12.8 (8.1)	15.3 (12.6)	15.8 (14.4)	7.1 (7.7)
